# Profiling of Androgen Response in Rainbow Trout Pubertal Testis: Relevance to Male Gonad Development and Spermatogenesis

**DOI:** 10.1371/journal.pone.0053302

**Published:** 2013-01-03

**Authors:** Antoine D. Rolland, Aurélie Lardenois, Anne-Sophie Goupil, Jean-Jacques Lareyre, Rémi Houlgatte, Frédéric Chalmel, Florence Le Gac

**Affiliations:** 1 INRA, UR1037 LPGP, SFR Biosit, Biogenouest, Campus de Beaulieu, Rennes, France; 2 Inserm, U1085, IRSET, Université de Rennes I, Campus de Beaulieu, Rennes, France; 3 Inserm, UMR1087, l’institut du thorax IRT - UN, Nantes, France; 4 Université de Nantes, Nantes, France; Baylor College of Medicine, United States of America

## Abstract

The capacity of testicular somatic cells to promote and sustain germ cell differentiation is largely regulated by sexual steroids and notably androgens. In fish species the importance of androgens is emphasized by their ability to induce sex reversal of the developing fries and to trigger spermatogenesis. Here we studied the influence of androgens on testicular gene expression in trout testis using microarrays. Following treatment of immature males with physiological doses of testosterone or 11-ketotestosterone, 418 genes that exhibit changes in expression were identified. Interestingly, the activity of testosterone appeared stronger than that of 11-ketotestosterone. Expression profiles of responsive genes throughout testis development and in isolated germ cells confirmed androgens to mainly affect gene expression in somatic cells. Furthermore, specific clusters of genes that exhibit regulation coincidently with changes in the natural circulating levels of androgens during the reproductive cycle were highlighted, reinforcing the physiological significance of these data. Among somatic genes, a phylogenetic footprinting study identified putative androgen response elements within the proximal promoter regions of 42 potential direct androgen target genes. Finally, androgens were also found to alter the germ line towards meiotic expression profiles, supporting the hypothesis of a role for the somatic responsive genes in driving germ cell fate. This study significantly increases our understanding of molecular pathways regulated by androgens in vertebrates. The highly cyclic testicular development in trout together with functions associated with regulated genes reveal potential mechanisms for androgen actions in tubule formation, steroid production, germ cell development and sperm secretion.

## Introduction

The hypothalamic-pituitary-gonadal axis regulates sexual maturation and spermatogenesis through the release of two gonadotropins from the anterior pituitary upon GnRH stimulation, the luteinizing hormone (LH) and the follicle-stimulating hormone (FSH) [1]. The control of spermatogenesis by gonadotropins mainly relies on LH-induced testosterone (T) production by Leydig cells, as observed in mammals: T is indeed sufficient to restore complete spermatogenesis in hypogonadal mice [2],[3] or hypophysectomized rats [4], [5] whereas FSH promotes Sertoli cell proliferation and differentiation and is thus responsible for the final spermatogenetic capacity of the adult testis [6], [7].T binds to the nuclear androgen receptor (AR) that, upon homodimirization, regulates expression of genes containing *cis* regulatory elements termed Androgen Response Elements (AREs). In mammals, AR is expressed in Leydig, peritubular and Sertoli cells in the adult testis [8]. Consequently, the control of spermatogenesis by T is more likely to occur by modulating somatic signaling rather than by a direct effect on the germ cells. This was further evidenced by the ability of AR^−/−^ spermatogonial stem cells to colonize recipient testes [9] and by the spermatogenetic defects observed in selective AR knockout in testicular somatic cells [10], [11], [12]. A number of studies have addressed the specific question of testicular gene modulation by androgens and, despite the use of various models (for review, see [13]), the way T regulates spermatogenesis are still not fully understood. Notably, only a few direct androgen-target genes have been identified so far and the specific role of T at specific steps in male gonad development is still unclear. In this regard, seasonally-breeding fish species such as the rainbow trout, *Onchorynchus mykiss*, represent an interesting model to investigate those molecular pathways regulated by androgens in the testis. The seasonal spermatogenetic cycle, together with the cystic organization of the trout testis, tends to synchronize the developmental state of somatic cells at any time. On the other hand, the endocrine landscape in teleost fish species appears somehow more complex than in mammals. In addition to T and androstenedione (Δ4) several 11-oxygenated androgens such as 11β-hydroxyandrostenedione (11βOHA), 11-ketoandrostenedione (11KA), 11β-hydroxytestosterone (11βOHT) or 11-ketotestosterone (11KT) are found in males and exhibit distinct plasma profiles during fish development and throughout the reproductive cycle [14]. Much evidence points towards 11KT being a potent androgen for promoting spermatogenesis in a few fish species [15], [16], [17], [18], [19]. Contradictory results were obtained for T in this regard: A direct stimulatory effect was reported on spermatogenesis in the Mummichog and goldfish [20], [21] whereas it was found to inhibit the 11KT-induced spermatogenesis in the African catfish *in vivo* [22].In this study we investigated the influence of androgens on gene expression in the trout testis at the onset of puberty. Immature males were submitted to short-term supplementation of either T or 11KT and testicular androgen-responsive genes were identified using microarrays. The use of expression data from testes at various developmental stages [23] further allowed identifying relevant genes that exhibit differential expression coincidently with changes of androgen circulating levels along the reproductive cycle. In addition, it enabled us to distinguish between the somatic or germ cell origin of responsive genes. Functions associated with regulated genes reveal potential mechanisms for androgen actions. Finally, we performed a computational phylogenetic footprinting analysis and pointed out potential direct target genes of androgens whose promoters harbour evolutionary conserved AREs.

## Materials and Methods

### Ethics Statement

Experimental research on animal reported here was performed in conformity with the principles for the use and care of laboratory animals in compliance with French and European regulations on animal welfare. Furthermore, experimenters were delivered an authorization given by the French “Direction des Services Vétérinaires” to conduct or supervise experimentations on live animals.

### Animals and Hormonal Treatment

Rainbow trout (*Oncorhynchus mykiss*) were maintained under natural temperature and photoperiod at the INRA experimental fish farm (Drennec, France).

Prepubertal male trout weighting 150+/−34 g were submitted to androgen supplementation using subcutaneous implants (Innovative Research of America). Pellets used as implants were a ready-to-implant form, using Matrix-Driven Delivery (MDD) Pellet System (>21 day release; Innovative Research of America, USA). Trout were anesthetized using phenoxyethanol and pellets were implanted in the dorsal muscle, just behind the dorsal fin, using a 10-gauge trochar. The control group (n = 14) received an empty pellet.

Four androgen supplementations were realized : 2 doses of testosterone (T) were compared to detect potential dose response effects. For the lowest T dose (T1 = 0.1 mg or 0.666 µg/g body weight; n = 5), a single time point (day 7) was investigated. For the highest T dose (T2 = 0.2 mg or 1.333 µg/g body weight ), 2 time points were investigated to eventually discriminate between early and late responses (day 7, n = 4; day 14, n = 5). The response to 11KT, a major androgen in fish physiology with low metabolisation potential, was also studied, although only at one dose and one time point (0.25 mg or 1.667 µg/g body weight, day 7; n = 7) for which the highest response to T was obtained.

Following supplementations, the testes were recovered, weighed to determine the gonadosomatic index (GSI) and rapidly immersed in Bouin’s solution for histological study or frozen until use for microarray analysis. Blood samples were collected and circulating androgen levels were measured by specific radioimmunoassay (RIA) as previously described [24]. These experiments were conducted in January and February, when most animals were immature (containing spermatogonia only in terms of germ cells). The testicular histology of both treated and control animals was also carefully analysed.

### cDNA Microarray Experiment

#### Microarray hybridization and raw data production

cDNA microarrays (platform: http://www.ncbi.nlm.nih.gov/geo/query/acc.cgi?acc=GPL3650) were generated by CRB GADIE (http://crb-gadie.inra.fr/) as previously described [25]. Procedures for RNA extraction, cDNA target synthesis using [alpha-33P] dCTP, microarray hybridization and raw data production have been previously described in details [23].

#### Normalization procedure

Expression data were normalized as previously described [23]. Briefly, raw data were corrected for the amount of spotted cDNA (Si/Vi). To avoid the bias affecting relative gene expression levels, the corrected signal of each spot was further multiplied by the median vector signal of all arrays for this same spot ((Si/Vi) x medVi). Expression values were then log2-transformed and submitted to a quantile-quantile normalization [26] using the AMEN software (http://sourceforge.net/projects/amen/; [27]. Raw data as well as a normalized expression file are available at the GeneOmnibus public data repository (http://www.ncbi.nlm.nih.gov/geo/query/acc.cgi?acc=GSE16030).

#### Statistical and cluster analyses

Non-informative clones for which too little cDNA was spotted (oligonucleotide signal <3 times the background level in more than 20% of samples) were removed from the analysis. Androgen-responsive genes were then identified by comparing the control group to each of the treated groups (T1, “T2, day7”, “T2, day14”, 11KT) using the multi-class Limma statistical test (FDR 1%) [28]. All differentially-expressed transcripts were submitted to the PAM algorithm (Euclidian distance measure) and grouped into 4 expression clusters. Three pairwise comparisons were also performed to identify genes differentially-regulated by testosterone and 11KT at a comparable time point (*i.e.* at day 7) using the Limma statistical test: control vs “T2, day7”, controls vs 11KT, 11KT vs “T2, day7”. Here, a Bonferroni adjustment was used to obtain a global risk of type I error equals to 1%. Thus, for any one comparison to be considered significant, the p-value had to be less than 0.0033, that is 1% divided by the number of pairwise comparisons. For heatmap representations, unlogged data were loaded into the MeV software (www.tm4.org/mev/) and normalized according to the following formula: Value = [(Value) – Mean(Row)]/[Standard deviation(Row)].

#### Metaanalysis

Expression data from testes at different developmental stages and isolated germ cells populations [23] were used to investigate the developmental pattern and the cell type in which androgen-responsive genes are expressed. Briefly, samples in this dataset included - Testes in early stages containing slowly-dividing type A spermatogonia (Stage I) or growing numbers of actively-dividing type B spermatogonia (Stages IIa and IIb) - Maturing testes also containing meiotic spermatocytes (Stage IIIb) and post-meiotic spermatids (Stage V) - Spawning testes containing essentially mature spermatozoa (stage VIII) (see [29], [30] for a more precise description of all stages) - Fractions of isolated germ cells enriched in spermatogonia, spermatocytes or spermatids obtained as previously described [31]. Genes differentially-expressed during testis development (F’s statistic permutation test, FDR 5‰) were compared to androgen-responsive genes.

### Trout Microarray Annotation and Functional Data Mining

Trout cDNAs spotted onto the microarray were annotated as previously described [23]. GeneOntology (GO) terms associated to fish genes and their rat, mouse and human orthologs were extracted from the Ensembl database (version 52). Biological mining of expression clusters was then performed by searching for over- and under-represented functional GO terms (Gaussian hypergeometric test) as compared to all well-measured genes, using the AMEN software.

### Prediction of Androgen Response Elements

As there is yet no genome sequence available for the *Oncorhynchus mykiss* or any other salmonid we used that of *Gasterosteus aculeatus* (Stickleback) as a reference. Stickleback Ensembl genes were identified as previously described [23] and their Transcriptional Start Sites (TSS) were localized using the *ensGene.txt* file from UCSC (http://genome.ucsc.edu/) [32]. AREs were predicted within genomic regions from −10000 base pairs (bp) up to +2000 bp with respect to each TSS. Validated ARE motif matrices (M00447, M00481, M00953, M00956 and M01201) from the Transfac Professional database (release 2009.1; http://www.biobase-international.com) [33] were predicted using the MATCH program [34]. Core and matrix similarity cutoff values were set at 0.8.To reduce the number of false positive and focus on motifs that are more likely to be functional, hits sequences were required to be conserved between species [35]. For each predicted motif, a cross-species conservation score was processed by averaging the base-by-base phasCons scores calculated between 8 vertebrates (chicken, fugu, human, medaka, mouse, stickleback, tetraodon, and zebrafish) as provided by the UCSC genome browser [36] and only motifs with such a cross-species conservation score ≥0.8 were considered. Similarly, we also screened for the presence of putative Estrogen Response Elements (EREs) in the same gene promoter regions using the validated M00191 Transfac matrice.

### qPCR Experiments

Total RNAs were first treated with the turbo DNA-*free*™ kit (Ambion) and 2 µg were submitted to reverse-transcription (RT) using random hexamer primers and 200 units of MMLV reverse transcriptase (Promega). Real-time PCR assays were performed on the StepOne™ Real-Time PCR System (Applied Biosystems) using 1∶30 diluted RT products, primers (300 nM) and Fast SYBR® Green Master Mix (Applied Biosystems). Relative expression levels were normalized using a reference gene, *rps15* (clone 1RT58B15_B_A08), designed on the basis of its invariant expression (low standard deviation) both in androgen treatment and in spermatogenesis development microarray experiments (Figure S1). Real-time PCR oligonucleotide primers were designed using the perlprimer software (http://sourceforge.net/projects/perlprimer/), verified with the oligoanalyser 3.1 web interface (http://eu.idtdna.com/analyzer/Applications/OligoAnalyzer/) to avoid self- and hetero-dimer formation as well as hairpin structures, and matched (BLAST algorithm) against the SIGENAE trout contig collection (som.8 version) to avoid non-specific annealing to other transcripts (Table S1). Aberrant values, as determined by Dixon test (p<5%), were removed and statistical analyses were performed using the Statistica software using the non-parametric test of Mann & Whitney (n individuals = 5 to 7).

## Results

### Short-term Supplementations of Immature Male Trout Induce Physiological Androgen Circulating Levels

We treated prepubertal males in early stages of maturation (stages I-II), for which low basal androgen levels (1 to 2 ng/ml) should make the detection of responsive genes more convenient and sensitive, and we used implants with moderate releasing rates so that possible toxicological effects would be very limited.

**Figure 1:**
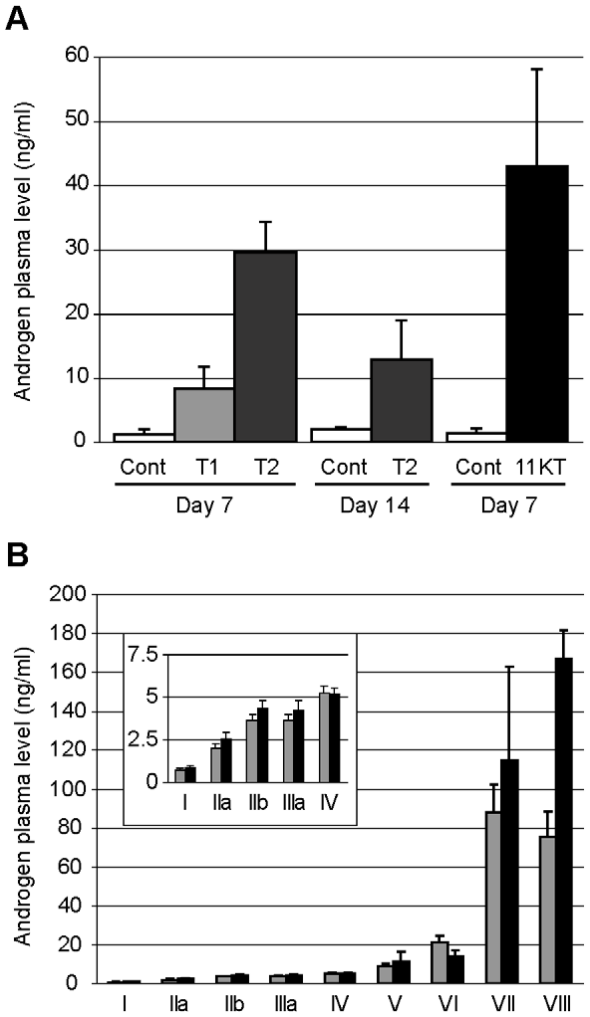
Androgen plasma levels in male trout. A : Increased androgen plasma concentrations following testosterone and 11-ketotestosterone supplementation. Androgen circulating levels were measured in stage I to II males supplemented for 7 or 14 days with testosterone (0.1 mg, T1; 0.2 mg, T2) or 11-ketotestosterone (0.25 mg, 11KT) as well as in corresponding controls (Cont). B : Natural testosterone (grey) and 11-ketotestosterone (black) circulating levels throughout the male trout reproductive cycle. Roman numerals (I-VIII) indicate testicular developmental stages. Mean values ± standard deviation are presented.

Supplementation of immature males with either T or 11KT resulted in a significant increase in corresponding blood plasma androgen concentrations (Figure 1A). T circulating levels were increased 8 and 28 times in animals treated for 7 days with low (T1) and high (T2) T doses, respectively (Figure 1A). Similarly, 11KT circulating levels were increased 32 times in animals treated for 7 days with this hormone as compared to controls (Figure 1A).

The induced androgen circulating levels correspond to physiological concentrations since natural T and 11KT circulating levels reach up to 90 and 170 ng/ml respectively during the trout reproductive cycle (Figure 1B). Additionally, by restricting the hormone supplementation to a few days, the gonadosomatic index of treated animals was unchanged and no striking change in cellular composition could be observed by qualitative histology (data not shown). These treatments were therefore found optimal to further investigating the influence of androgens on trout testicular gene expression.

### Identification of Androgen-responsive Genes in the Trout Testis

We first identified androgen-regulated genes by comparing testicular gene expression in controls and in all 4 treated groups. Among 7916 accurately measured cDNAs, 485 were differentially expressed (FDR <1%) in testes of supplemented animals (Figure S2, panel A). Overall the majority of these changes occurred through up-regulation. Expression signals of only 100 cDNAs were decreased following androgen treatment (cluster 1) whereas 327 were increased: 203 cDNAs showed the highest up-regulation at day 7 following T implantation (T2 showing generally more effects than the lower dose T1) (cluster 2) and 124 showed the highest induction at day 14 (cluster 3). In addition, a smaller cluster of 58 cDNAs exhibited down-regulation at day 7 and up-regulation at day 14 (cluster 4). Extensive information for all the androgen-responsive genes, including expression data, cluster information and corresponding annotations, is provided in File S1.

We next compared the effects of different androgen treatments at day 7 and found that the influence of T was stronger than that of 11KT: Among the 485 androgen-regulated genes, 84 genes were indeed significantly regulated by T only (T2, day 7) while 6 genes were regulated by 11KT only (p<0.0033) (Figure S3; these genes are marked “T only” and “11KT only” in File S1). In addition, all the 26 genes regulated by both androgens were found to be more influenced by T treatment (p<0.0033; Figure S3; marked “T >11KT” in File S1).

### Androgens Mainly Affect Gene Expression in Testicular Somatic Cells

The expression profiles of responsive genes were then further investigated during pubertal development of the gonad, using expression data obtained from trout testes in different maturation states (early stages I to V of spermatogenesis and late stage VIII of sperm excretion in the efferent ducts) and from isolated germ cell fractions enriched in spermatogonia, spermatocytes or spermatids [23].

**Figure 2:**
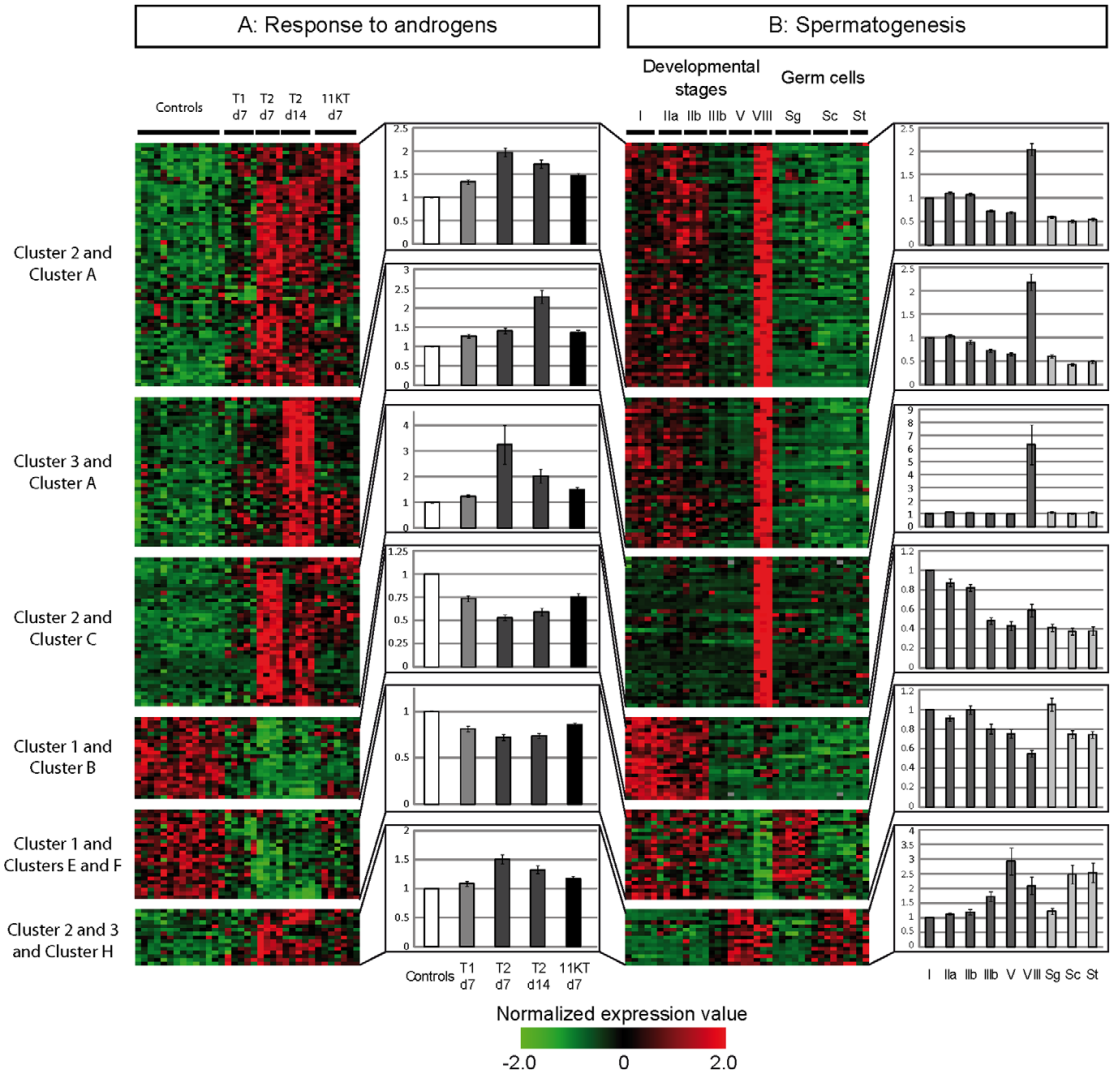
Expression of androgen-responsive and developmentally-regulated genes. Heatmap representation of specific expression clusters of androgen-regulated genes that also display differential expression during trout spermatogenesis. Subgroups of genes were established by crossing 4 clusters (1 to 4) of androgen-responsive genes and 9 clusters (A to I) of spermatogenesis as indicated in table 1. For these genes, we present expression signals obtained: - in testes from controls and androgen-supplemented animals, and - in testes at various developmental stages and in enriched fractions of isolated germ cells. Normalized expression values are shown according to the scale bar while histograms represent averaged fold changes to the control (androgens data) or to the stage I (spermatogenesis data) ± SEM for each cluster. T1 and T2 correspond to animals supplemented with testosterone implants of 0.1 and 0.2 mg, respectively; 11KT indicates animals treated with 11-ketotestosterone implants of 0.25 mg; d7 and d14 correspond to animals treated for 7 and 14 days, respectively. Roman numerals (I–V and VIII) indicate testicular developmental stages. Sg, Sc and St correspond to isolated germ cell populations enriched in spermatogonia, spermatocytes and spermatids, respectively. The annotation of each gene is accessible in the searchable File S1.

Consistent with a physiological response to androgen supplementation we found that most of the androgen-responsive genes were also differentially expressed during the course of the reproductive cycle (Figure S2, panel B). More importantly, we showed that androgen-responsive genes were preferentially expressed in somatic cells: Among the 304 androgen-regulated genes that were identified as differentially-expressed during spermatogenesis, 242 were indeed found in somatic expression clusters (A–D in table 1) while only 62 belonged to germ cell expression clusters (E–I in table 1) (Figure 2).

**Table 1:** Summary of androgen-responsive clones that also exhibit differential expression during spermatogenesis.

	1 - Downregulated	2 - Upregulatedat day 7	3 - Upregulatedat day 14	4 - Down at day 7, Up at day 14
A - Somatic	4	**65**	**40**	6
B - Somatic, low in stage VIII	**22**	5	**22**	9
C - Somatic, high in stage VIII	1	**40**	6	3
D - Somatic+isolated spermatogonia	2	7	7	4
E - Spermatogonia - type A	**19**	1	1	4
F - Spermatogonia - type B	**5**	2	2	1
G - Germline	2	3	2	0
H - Meiotic/Post-meiotic	0	**10**	**5**	1
I - Isolated germ cells only	1	3	0	0

Subgroups of clones were established by crossing 4 clusters of androgen-responsive genes (1 to 4, present study) and 9 clusters (A to I) of genes differentially expressed during trout spermatogenetic development [23]. Bolded numbers indicate subgroups of genes that are represented in Figure 2.

Strikingly, most up-regulated genes were found in the “somatic” clusters A or C of the spermatogenesis study (Table 1 and Figure 2), which exhibit a high or a very high expression in stage VIII testes, coinciding with the greatest circulating androgen levels during the reproductive cycle (Figure 1B). Conversely, out of the 29 somatic genes that were down-regulated following experimental androgen supplementation (cluster 1), 22 were found in cluster B of the spermatogenesis study, which exhibits low or very low expression in stage VIII testes (Table 1 and Figure 2).

The dynamics of these somatic genes are therefore highly consistent with a regulation by endogenous androgens during the natural course of the reproductive cycle, and with a role in testis maturation or in the spermatogenetic processes. Important to note is that, while T and 11KT exhibit highest circulating levels during late phases of the reproductive cycle, they first significantly increase at the recrudescence phase (Stages I-II-III, panel in Figure 1B). Therefore part of the responsive genes may be tightly regulated by androgens at the onset of spermatogenesis.

Importantly, a few genes from the germ cell expression clusters also exhibited remarkable features following androgen supplementations : Out of the 35 androgen-responsive genes from “spermatogonial” expression clusters (E and F), 24 were down-regulated in treated animals (cluster 1). On the other hand, 15 of the 16 genes from the meiotic/post-meiotic expression cluster (H) were up-regulated (clusters 2 and 3) following androgen treatment (Table 1 and Figure 2). This indicates that, in addition to regulating gene expression in supporting somatic cells, androgen treatment would result in a shift in germ cell expression profiles, from mitotic spermatogonia to meiotic spermatocytes.

### Potential Direct Targets of Androgens are Revealed by Promoter Analysis

Like in most studies that aimed at identifying androgen-target genes in the testis, the picture obtained in our experimental scheme is likely to result from a complex cascade of gene, protein or endocrine regulations, with only a small number of genes being under the direct control of the AR.

**Figure 3:**
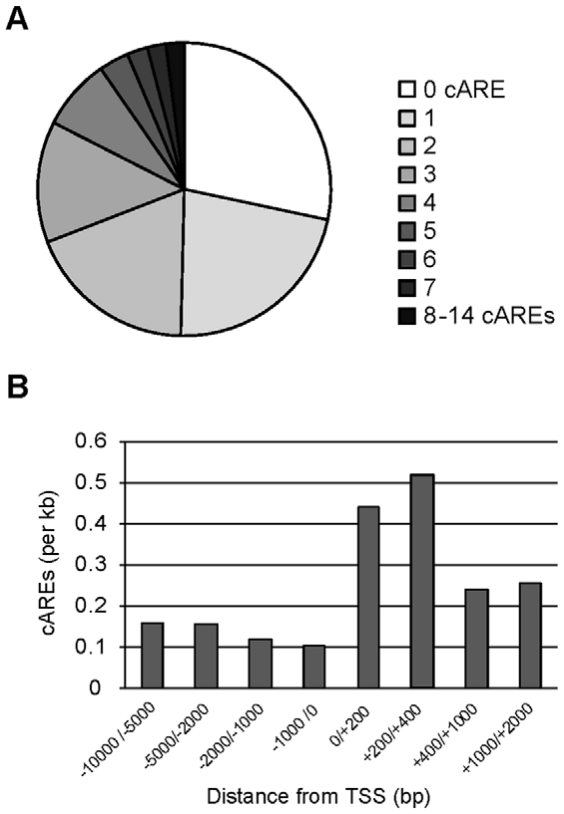
Identification of conserved Androgen Response Elements within the promoter of androgen-regulated genes. A : The relative number of genes displaying 0 to 14 conserved AREs is presented. Conserved AREs (cAREs) were searched in a region of −10000/+2000 base pairs relative to the TSS in the promoter of 395 androgen-responsive gene orthologs in *Gasterosteus aculeatus*. B : Frequency of conserved AREs in the promoter regions of androgen-responsive genes with respect to the TSS, expressed by number of sites per kilobase of DNA.

To identify genes potentially directly regulated by androgens we searched for AREs within the promoter of androgen-regulated genes. Because the *Oncorhynchus mykiss* genome was not available we used that of *Gasterosteus aculeatus* as a reference and considered evolutionary conserved AREs only (i.e. sequences with a high evolutionary conservation score across up to 8 vertebrates). A total of 775 matching sequences were assigned to the promoter regions (−10000/+2000 bp relative to the TSSs) of 283 out of the 395 trout androgen-regulated genes that also displayed a clear ortholog in *Gasterosteus aculeatus* (Figure 3). We next focused on those androgen responsive genes that also exhibit differential expression during spermatogenesis and, when restricting the exploration to a more proximal promoter (−2000/+400 bp relative to the TSS), we identified 53 genes (18.7%) that presented at least one putative conserved ARE. We found that 42 of these genes belonged to somatic cell expression clusters, which represent the most promising direct targets of the AR in this study (column labeled “predicted as “Somatic” with a proximal ARE” in File S2).

Of note is that searching for conserved AREs was compulsory for us in order to reveal AREs that might also be present in the trout genome. While this method fails to identify a subset of species-specific regulatory elements, it allows focusing on sequences that are more likely to be functional given that important DNA regulatory regions tend to be conserved between species [35]. All information regarding androgen-responsive genes that contain at least one conserved ARE in their promoter regions (−10000/+2000 bp relative to the TSSs) is reported in the File S2.

### Functional Implication of Androgens are Revealed by GeneOntology Terms Analysis

**Figure 4:**
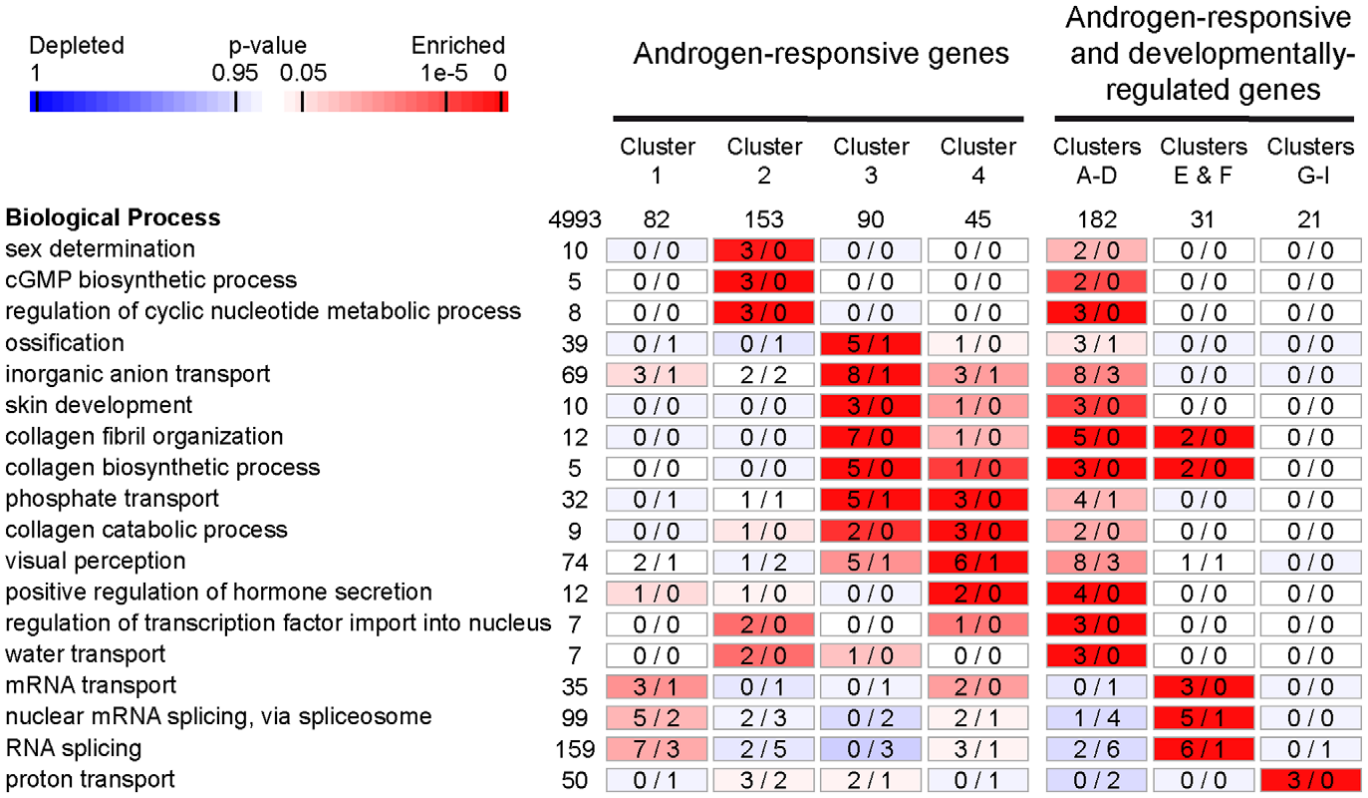
Enriched biological processes among testicular androgen-responsive genes. Over-represented “biological process” terms from the GeneOntology (GO) were identified in the 4 expression clusters of androgen-responsive genes (1 to 4) as well as in 3 groups of androgen-regulated genes that exhibit “somatic” (A to D), “spermatogonial” (E and F) and “germline” (G to I) expression profiles during spermatogenesis. Rectangles indicate the observed (left) and expected (right) numbers of genes bearing the corresponding GO term whereas the number of genes exhibiting this GO term on the entire microarray is given on the left. Only GO terms with a p-value of ≤10^−6^ and for which at least 3 non-redundant genes were in the cluster were considered as statistically enriched. To avoid redundancy between closely related terms an Ontology Specific Information Rate (OSIR) cutoff of ≥0.95 was selected [27]. Numbers in bold indicate a statistical enrichment for a given GO term according to the scale bar.

We performed a GeneOntology (GO) term analysis to evaluate the functional impact of testicular changes in expression mediated by androgens (Figure 4 and Figure S4).

Importantly a statistical enrichment was found for genes involved in “sex determination” within genes that were up-regulated mainly at day 7 (cluster 2). The same cluster was enriched in genes involved in “regulation of cyclic nucleotide metabolic process”. Genes that were up-regulated later on, at day 14 (clusters 3 and 4), were found to bear common and overlapping functions related to “collagen metabolism” and “collagen organization”, “ion transport” or several developmental processes such as “ossification”, “skin development” and “visual perception” (Figure 4). When focusing on androgen-regulated genes with “somatic” expression (clusterA–D), additional processes were also found enriched such as “positive regulation of hormone secretion” “regulation of transcription factor import into nucleus” or “water transport”.

Overall these particular expression changes suggest an important role for androgens in remodelling extracellular matrix and the seminiferous tubule structure, which was also evidenced at the “Molecular function” (e.g. “extracellular matrix structural constituent”, “structural molecule activity”) and “Cellular component” levels (e.g. “extracellular region” and “extracellular matrix part”) (Figure S4).

### Real-time PCR Validation of Candidate Genes

**Figure 5:**
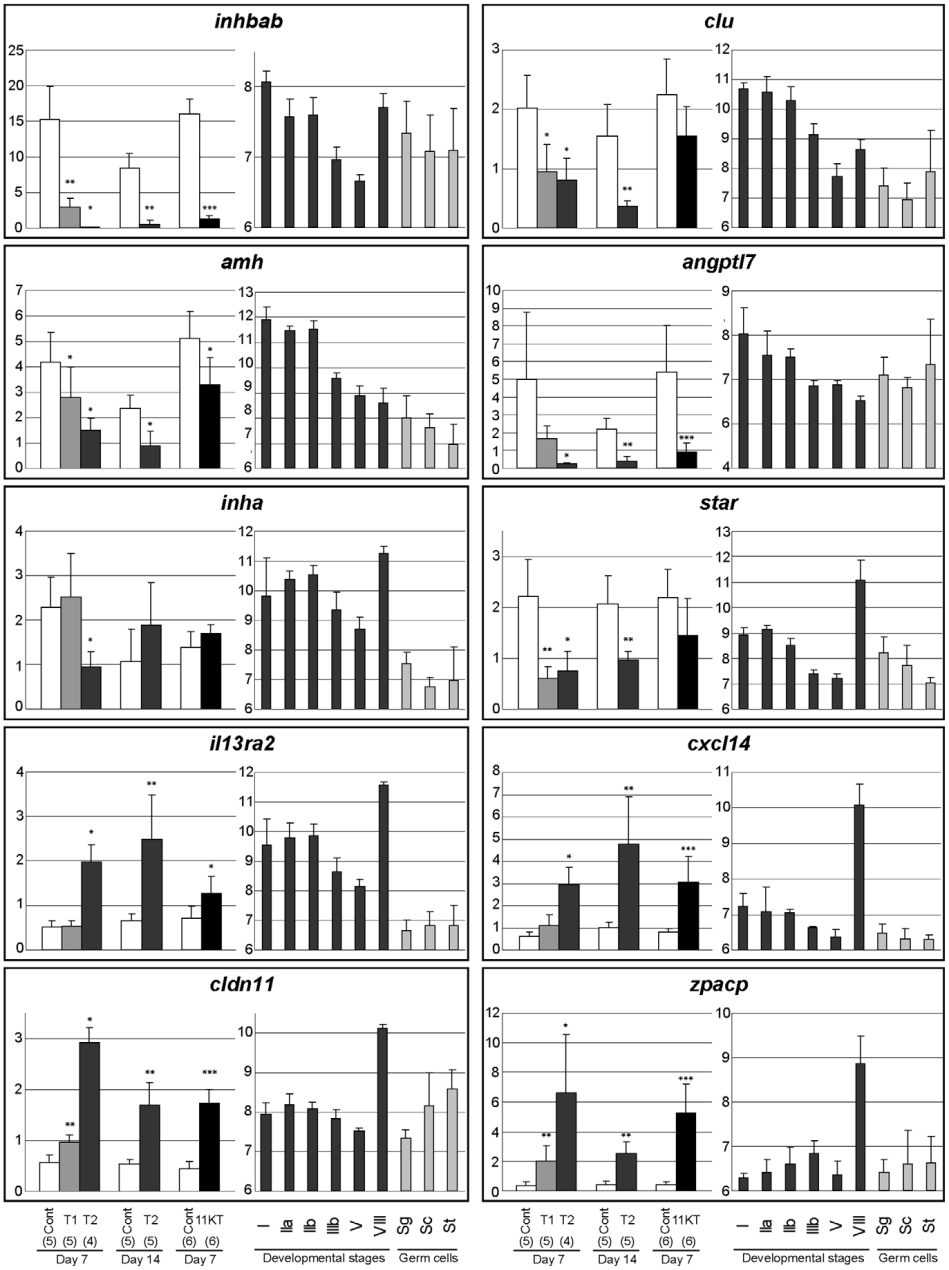
Real-time PCR validation of candidate genes. Relative expression profiles as measured by qPCR in the androgen supplementation experiment (left panel) and by microarray during spermatogenesis (right panel). The mRNA levels measured by qPCR were normalised to the Rps15 gene reference levels whereas microarray data correspond to Log2-transformed signal intensities. Mean values ± SEM are are presented. One, 2 or 3 star symbols indicate p<0.05, p<0.01 or p<0.005, respectively, when comparing treated samples and corresponding controls (cont) using the non-parametric test of Mann & Whitney. T1 and T2 correspond to animals supplemented with testosterone implants of 0.1 and 0.2 mg, respectively. 11KT indicates animals treated with 11-ketotestosterone implants of 0.25 mg. Day 7 and Day 14 correspond to animals treated for 7 and 14 days, respectively. The number of biological replicates used for qPCR experiments is indicated in brackets. Roman numbers (I–V and VIII) indicate testicular developmental stages analysed by microarrays. Sg = spermatogonia. Sc = spermatocytes. St = spermatids.

We choose several somatic transcripts possibly involved in paracrine regulations and representative of the 3 major androgen-responsive clusters to perform qPCR measurments (Figure 5). We confirmed the strong inhibition of 4 genes that are also down-regulated during the spermatogenetic maturation process: *inhba* and *clu* are inhibited by both T and 11KT whereas *amh* and *angptl7* are mainly influenced by T. In addition, the moderate down-regulation of *inha* following 7 days of T supplementation and the strong and wide inhibition of *star* by T were also confirmed. Noteworthy is that these 2 genes exhibited high expression in stage VIII testes. Among the up-regulated genes we investigated, *il13ra2*, *cxcl14*, *cldn11* and *zpacp* were confirmed to be increased by T and 11KT (Figure 5), while the up-regulation of *fgf12* was confirmed statistically for 11KT only (data not shown).

We also intended to validate the regulatory effect of androgen treatments on several somatic genes encoding for transcription factors of interest for testis pubertal development (Figure 5). *tbx1*, whose expression strongly decreases throughout the reproductive cycle, was confirmed to be down-regulated, while *dmrt1*, a testis-specific factor, was up-regulated by the two androgens. Surprisingly, when investigating the expression of *sox9*, only *sox9a* was confirmed to be highly up-regulated after both T and 11KT treatment whereas *sox9b* was down-regulated by T. It is likely that *sox9a* mRNA actually cross-hybridizes onto the *sox9b* cDNA probe, resulting in its apparent up-regulation of both factors when using cDNA microarrays.

**Figure 6:**
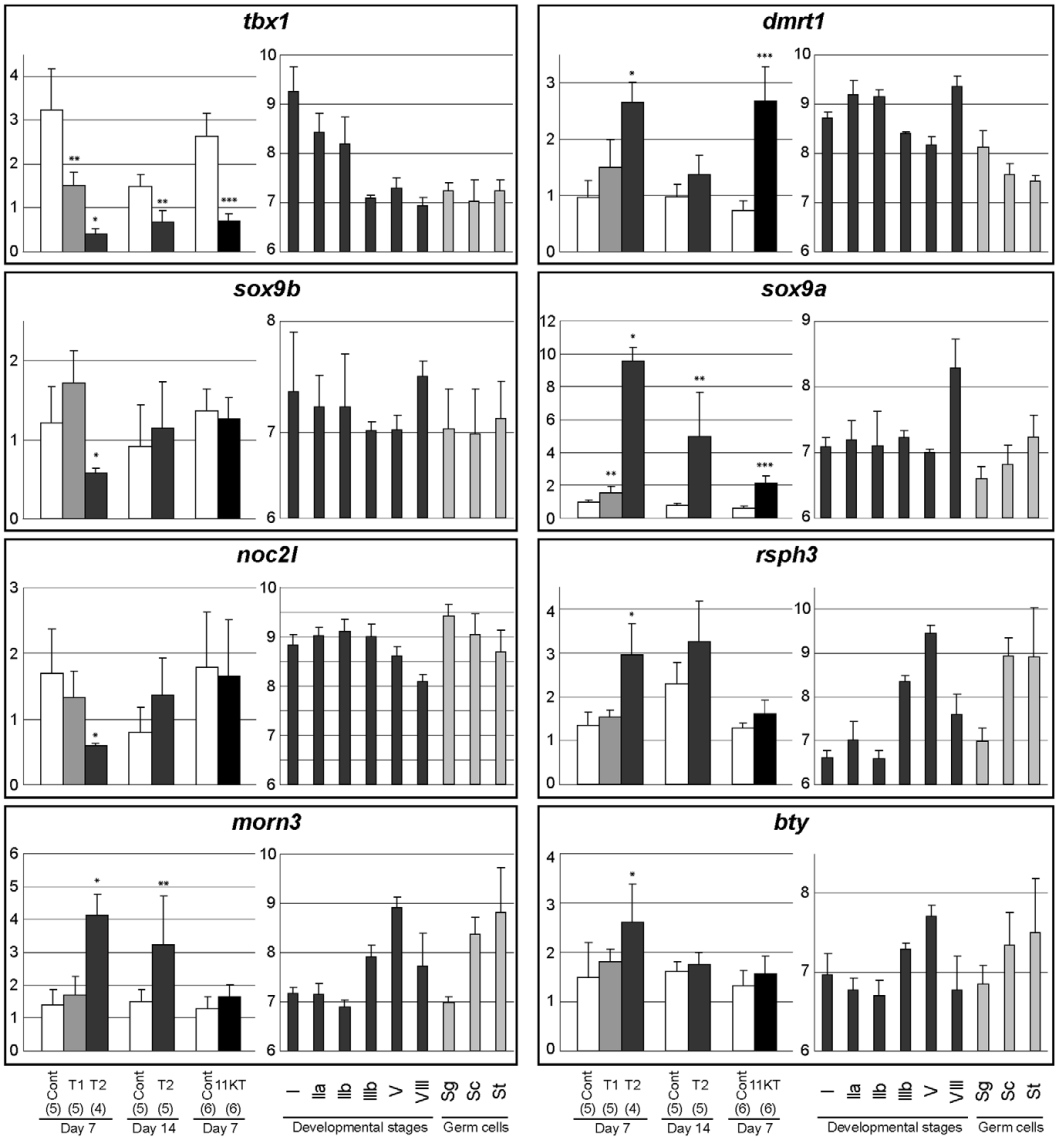
Real-time PCR validation of candidate genes (continued).

The expression of 8 germ cells transcripts was also further investigated. In those cases the statistical validation was rendered difficult post probably because of the low amplitude of androgen effects, notably for genes preferentially expressed in spermatogonia and down-regulated by androgens (*noc21*, *bop1*, *eprs*, *prpf8* and *rnf17*). However, similar profiles for PCR and microarray data were obtained for *eprs* and *prpf8* (data not shown) and a significant down-regulation was confirmed for *noc2l* (Figure 6). Furthermore 3 genes preferentially expressed in meiotic and post-meiotic germ cells, *rsph3*, *morn3* and *bty*, were confirmed to be up-regulated following T supplementation.

## Discussion

Androgens, in fish species notably, have been shown to masculinize the female gonad, to trigger spermatogenesis onset and to promote testis growth and maturation. In this study we provide new information on molecular pathways regulated by androgens in the testis of a vertebrate.We identified a complement of genes showing transcript regulation in fish testis after androgen supplementation, and a number of evidences support the physiological relevance of the data: Changes in expression were obtained in response to physiological doses of androgens and before any modification of the testis cell composition could be detected. The highly cyclic and partially synchronous development of the trout testis made it a choice model to show that many of these androgen-regulated genes are naturally differentially expressed at particular stages of testicular development, suggesting that they are indeed involved during testis maturation or during spermatogenesis. The model also enabled to predict in which cell type the regulated transcripts are expressed [23]: Regulated transcripts were preferentially found in the somatic compartment of the gonad, which is in agreement with the known expression of the AR in testicular somatic cells ([37], [38]; and this work: Figure S5). We notably find many somatic cell genes whose developmental patterns fit well with changes in endogenous androgen circulating levels during natural testis maturation, which further reinforces the physiological significance of the data reported here. With this study we thus provide a substantial body of data and identify genes that likely contribute to pubertal development in the testis. Most of the comprehensive and useful information regarding these androgen-regulated genes - including expression during testis development, promoter analysis as well as annotation - is provided in the File S1 and in the File S2. Taken together these data also allow further speculating on the molecular mechanisms of androgen action in the testis.

The potential impact of androgens could include an active role in the initiation of spermatogenesis. Indeed, besides their effects on somatic cells, androgens altered the germ line towards more differentiated germ cell expression profiles. This would be quite in agreement with experimental data obtained in other fish species that showed androgens to induce spermatogenesis or accelerate this process (for reviews, see [39], [40]). In trout, Baron and collaborators found that in early sexual differentiation, a prolonged treatment of female embryos with high doses of androgens promote testicular organization with apparition of meiosis whereas control male gonads contain only spermatogonia [41]. The way androgens act on germ cells and influence their proliferation and differentiation is likely to be indirect and to involve growth factors and extracellular signaling from the somatic compartment. Importantly, we found that androgens strongly down-regulate *inhba* and *amh* transcripts, two members of the Activin family. Activins have effects on many physiological processes, including a stimulatory effect on spermatogonia proliferation [42], [43]. Inhibition of the ßA subunit would prevent the formation of biologically active activin, particularly activin A (dimer ßA-ßA), and could therefore have a blocking action on germ cell proliferation and allow their differentiation towards meiosis. This is consistent with our observation of a rapid decrease of this transcript when meiosis develops during the reproductive cycle (stages III-V of spermatogenesis), coincidently with an increase in androgen levels. AMH (or Mullerian inhibiting substance) production in the mouse fetal testis has been linked with male-specific differentiation events associated with the assignment of male gender to PGCs [44]. *amh* expression and its hormonal regulation were studied in trout during gonad sex differentiation [45] but its role in the adult male gonad is mainly ignored. In vitro, when applied to prepubertal eel and zebrafish testis explants, AMH prevents androgen-stimulated proliferation or differentiation of early spermatogonia [46], [47]. For the first time in trout we show that both T and 11KT can down-regulate *amh* in the early stages of pubertal maturation. Interestingly, we show that *amh* transcript relative abundance rapidly decreases during the natural reproductive cycle in inverse correlation with the large increase in circulating levels of T and 11KT (this study), and that *amh* transcripts are preferentially accumulated in Sertoli cells surrounding early spermatogonia [23]. In agreement, accumulation of the *ar* transcripts in Sertoli cells surrounding early germ cells was demonstrated in zebrafish testis [37]. Our data reinforce the idea that androgen-mediated suppressions of AMH and activin are physiologically required for initiation of active spermatogenesis at puberty. We propose to extend this hypothesis to several down-regulated candidates from cluster 1 with known functions in cell fate/growth or differentiation, including inhibin, folistatin like 1, BMP and activin membrane-bound inhibitor (BAMBI), Platelet-derived growth factor receptor beta, TGFB-inducible early growth response protein 2, Proliferation-inducing gene 32 protein, secreted retinol binding protein 4 and Angiopoietin-like factor 7. Other genes involved in cell commitment and specification (*tbx1*, *sox8*, *sox9a* and *sox9b*), were found regulated in androgen-treated prepubertal testes. By regulating the expression of such genes, androgens might influence the differentiation and function of the supporting somatic cells during the reproductive cycle. Furthermore, some of these genes are known as important for sex determination/differentiation (*sox9a*, *dmrt1* and *nr0b1*) in the trout larvae [41], which emphasizes some similarities of androgen action both in adult testis development and in early gonad differentiation.

In addition to their roles during testis recrudescence, other regulated genes also emerge as probably involved in later stages of the reproductive cycle, at the time of sperm excretion. Within up-regulated genes, statistical enrichment was found for genes involved in “water transport” (*aqp1*, *aqp4* and *itpn*/*vspn*) and “ion transport”, including “chloride transport” and “potassium transport” (*c1qtnf5*, *c1qtnf6*, *clic4*, *col1a1*, *col5a1*, *col6a2*, *col12a1*, *col18a1*, *gabra1*, *gabra3*, *ppap2a*, *slc12a4* and *slc26a4*), and “proton transport” (*atp6v0a2*, *atp6v1f* and *rsph3*). These functions are highly relevant concerning the final maturation of spermatozoa in fish, which relies on seminal plasma pH and Ca2+ concentrations, their motility in the male tract, which depends on K+ concentration, and their excretion, which involves H2O transport [48], [49]. Strikingly, these genes were also found strongly up-regulated in the ultimate stages of normal testicular development or spermatogenesis (Figure 2: clusters A, C, G and H). Therefore the related genes reported here represent strong new candidates in sex steroid control of sperm hydration and final maturation in seminiferous tubules and efferent ducts.

An interesting issue regarding androgen function in fish species resides in the respective roles of T and 11KT for promoting male germ cell differentiation: 11KT was shown to be a potent androgen in some species [15], [16], [17], [19], and in other cases T was shown to have no, or only weak positive effects [18], [21]. More, T inhibits the positive effects of 11KT on germ cell proliferation and testis maturation in the African catfish [22]. Under our experimental conditions T appeared quantitatively and in several cases qualitatively more active than 11KT (more transcripts significantly modified with stronger regulatory effects following 7 days of treatment) at similar blood plasma concentrations. The mechanisms underlying the different effects of T and 11KT remain unknown. Specific conformation changes of the AR upon ligand binding may account for an efficient interaction with different transcription cofactors. Furthermore, the rainbow trout contains two distinct isoforms of the androgen receptor, ARα1 and ARα2 [50], [51], that may differ in terms of response to 11KT as suggested by the binding affinities of the ARα2 in the salmon ovary [52]. Importantly, part of the specific effects we observed for T may occur through its metabolisation into active products, either locally or peripherally, including aromatization into estradiol and the subsequent regulation of estrogen-responsive genes. However, no evidence of such estrogenic effects could be noticed: Indeed, according to unpublished microarray data, the genes responding to T did not demonstrate significant changes in estradiol-treated males (Figure S6). Actually, only two out of the 418 androgen-responsive genes were also found to be regulated following estradiol-supplementation. Conversely, at the time points analysed, androgen supplementations did not result in change in expression for the vitellogenin or the choriogenin L genes, two highly responsive genes to estrogenic compounds in many fish tissues, including the testis (Figure S6; [53]). These observations support the idea that T and 11KT have both distinct and common functions in the gonad, even though a more detailed analysis (several time points and several doses) would be required to make final conclusions on the potencies of T compared to 11KT.

Although we presume that most changes in expression occurred via the intra-testicular action of androgens, we cannot exclude that the effects we observe after *in vivo* supplementation partially result from alteration in the circulating levels of gonadotropin or other endocrine factors, or from the regulation of the testicular hormone receptivity. Indeed, positive and inhibitory effects on basal gonadotropin secretion by testosterone during the early-recrudescence phase of the gonadal cycle have been reported [54], [55]. In addition, we found that the *fshr* transcript was slightly down-regulated and that *lhr* was up-regulated at day 7, suggesting that the responsiveness to gonadotropin might be changed in treated animals. While the expression of *arα1* and *arα2* themselves was not modified following androgen treatment (Figure S5), genes encoding factors involved in the androgen or steroid metabolic pathways (*cyp17a1*, *fdxr*, *hs3db2*, *star* and *tdh*) or that mediate the response to androgens (*nr0b1* and *pap2a*) were regulated. This is consistent with the modulation of steroidogenesis in an auto-regulatory manner [56]. Such feedback loops are important for fine tune local regulation of testicular steroid production, and inhibitory effects may prevent Leydig cell hyperactivity in some stages of puberty.

Finally, when androgens act directly at the testis level, only a small proportion of the regulated genes are expected to be under the direct control of the AR. Using a phylogenetic footprinting approach we identified 42 genes as potential direct targets of the AR in the trout testis. Indeed, those androgen-regulated genes are of particular relevance as they have at least one highly conserved ARE predicted in their proximal promoter region (−2000/+400 with respect to their TSSs) and they are expressed in somatic cells where the AR is also present. This *in silico* analysis also highlighted genes that harbor two or more putative AR binding sites nearby the transcription initiation start site and are of great interest : Others and we have indeed demonstrated that multiple AREs, cooperate to confer full responsiveness and cell specific expression of some androgen target genes including for example, Igf1 [57], Lcn5 [58], Rhox5 [59] or Slp [60]. Of note is that, in addition to cAREs, we also found potential cEREs within the promoter of androgen-responsive genes (File S2). *In silico* promoter analyses indeed remain predictions and we we do not expect all predicted response elements to be functional. Instead we believe the somatic genes that respond to androgens and that also display proximal or multiple putative cAREs are preferential candidates for future functional validations.

In conclusion, we have identified a complement of genes expressed in the somatic compartment and predicted to be under the influence of androgens during testicular development in trout. Functions associated with these genes reveal potential mechanisms through which androgen exert their regulatory actions in tubule formation, germ cell differentiation, sperm secretion, and steroid production. Therefore we provide meaningful information on the mechanism by which T and/or 11KT could regulate germ cell production in vertebrates.

## Supporting Information

Figure S1 **Expression profile of the reference gene** ***rps15*** **in microarray datasets.** Expression of *rsp15* (clone 1RT58B15_B_A08) as measured in androgen supplementation and spermatogenesis datasets. Histograms represent means ± standard deviation of Log2-transformed signal intensities. Cont correspond to untreated control animals. T1 and T2 correspond to animals supplemented with testosterone implants of 0.1 and 0.2 mg, respectively. 11KT indicates animals treated with 11-ketotestosterone implants of 0.25 mg. Day 7 and Day 14 indicate 7 and 14 day post-implantation, respectively. Roman numbers (I–V and VIII) indicate testicular developmental stages. Sg = spermatogonia. Sc = spermatocytes. St = spermatids.
(TIF)

Figure S2 **Changes in testicular gene expression following androgen supplementation.** Heatmap representation of 418 androgen-responsive genes (485 clones) in the trout testis. After statistical filtration, co-expressed transcripts were classified into 4 clusters (1–4) using the PAM algorithm. For these genes, we present expression signals obtained: - in testes from controls and androgen-supplemented animals, and - in testes at various developmental stages and enriched fractions of isolated germ cells. Each line represents the expression signal of a single clone and each column is a sample. Normalized expression values are shown according to the scale bar while histograms represent averaged fold changes to the control (androgens data) or to satge I (spermatogenesis data) ± SEM for each cluster. T1 and T2 correspond to animals supplemented with 0.1 and 0.2 mg testosterone implants, respectively; 11KT indicates animals treated with 0.25 mg 11-ketotestosterone implants; d7 and d14 correspond to animals treated for 7 and 14 days, respectively. The gene annotation of each cluster is accessible in the searchable File S1.
(TIF)

Figure S3 **Expression of genes altered differently by testosterone and 11-ketotestosterone treatments.** Heatmap representation of 103 androgen-responsive genes (116 clones) which regulation by testosterone and 11-ketotestosterone differs statistically. Genes are displayed according to their response to testosterone only (T only; Up- or Down-regulated), to 11-ketotestosterone only, or to their greater response to testosterone (T >11KT; Up or Down regulated). Each line represents the expression signal of a single clone and each column is a sample. Normalized expression values are shown according to the scale bar while histograms represent averaged fold changes to the control ± SEM for each cluster. T1 and T2 correspond to animals supplemented with testosterone implants of 0.1 and 0.2 mg, respectively; 11KT indicates animals treated with 11-ketotestosterone implants of 0.25 mg; d7 and d14 correspond to animals treated during 7 and 14 days, respectively. The gene annotation of each cluster is accessible in the searchable File S1.
(TIF)

Figure S4 **Enriched molecular functions and cellular components GeneOntology terms among testicular androgen-responsive genes.** Over-represented “biological process” terms from the GeneOntology (GO) were identified in the 4 expression clusters of androgen-responsive genes (1 to 4) as well as in 3 groups of androgen-regulated genes that exhibit “somatic” (A to D), “spermatogonial” (E and F) and “germline” (G to I) expression profiles during spermatogenesis. Rectangles indicate the observed (left) and expected (right) numbers of genes bearing the corresponding GO term whereas the number of genes exhibiting this GO term on the entire microarray is given on the left. Only GO terms with a p-value of ≤10^−6^ and for which at least 3 non-redundant genes belonged to the cluster were considered as statistically-enriched. To avoid redundancy between closely related terms an Ontology Specific Information Rate (OSIR) cutoff of ≥0.95 was selected [27]. Bolded numbers indicate a statistical enrichment for a given GO term according to the scale bar.
(TIF)

Figure S5 **Expression profiles of** ***arα1*** **and** ***arα2.*** Expression of *arα1* and *arα2* was measured by qPCR and normalised to *rps15* expression levels. Cont corresponds to untreated control animals. T1 and T2 correspond to animals supplemented with testosterone implants of 0.1 and 0.2 mg, respectively. 11KT indicates animals treated with 11-ketotestosterone implants of 0.25 mg. Day 7 and Day 14 indicate 7 and 14 day post-implantation, respectively. Roman numerals (I-VIII) indicate developmental stages. SgA/B = Type A/B spermatogonia; ScI/II = primary/secondary spermatocytes; St = spermatids
(TIF)

Figure S6 **Androgen-** ***versus*** **estrogen-responsive genes.** A: Heatmap representation of the expression of all the androgen-responsive genes according to the androgen-supplementation experiment (this study) as well as to the estradiol-supplementation experiment (unpublished data). Normalized expression data are displayed according to the scale bar. B: Venn diagram showing the overlap between androgen-responsive genes (this study) and estradiol-responsive genes (unpublished data). C-F: Expression profiles (as determined by microarray analysis) for two estradiol-responsive genes (choriogenin in C, vitellogenin in D) and for two estradiol- and androgene-responsive genes (*aldh1l2* in E, *mfap2* in E). Histograms represent fold changes to the control ± SEM.
(TIF)

Table S1 **Sequences of primers used in qPCR experiments.** The clone name, the corresponding gene symbol and the sequence of forward and reverse primers (5′-3′) used for qPCR measurements are indicated.
(DOC)

File S1 **Androgen-responsive genes in the trout testis.** A searchable excel file containing normalized expression data (Log-2 transformed), annotations, and information about “Androgen response” (clusters 1 to 4), “Testicular development” (clusters A to I) and the identification of “conserved ARE” for the 485 androgen-responsive clones. Annotation provided contains the “Clone Name”, the “Annotation status” (“None”, “Not confident”, “Confident”), the “EST Name” and “Organism” retained for annotation, the corresponding “BLAT Score”, the “Matching type” (“Exonic”, “Intronic” or “Intergenic”), the “Overlapping length” (# base pairs; if <0 corresponds to the distance from the closest gene), the confidence index “n”, the “Fish ortholog Ensembl gene IDs” and “Fish ortholog Gene Symbols” and “Descriptions”, the “Non redundant ID” (Ensembl Gene ID of fish orthologs according to the following species availability: *Gasteosteus aculeatus*, *Danio rerio*, *Oryzias latipes* or *Takifugu rubripes*), the “Mammalian ortholog Ensembl gene IDs” (for human, mouse and rat), the “Mammalian ortholog gene symbols” and “Descriptions” and associated GeneOntology terms and IDs (“Biological process”, Molecular function” and “Cellular component”).
(XLS)

File S2 **Identification of genes with putative conserved Androgen Response Elements.** An excel file containing all putative conserved Androgen Response Elements (cAREs) and Estrogen Response Elements (cEREs) identified in the promoter of *Gasterosteus aculeatus* genes. Each line corresponds to one such putative ARE. For genes harbouring several of these putative response elements, as many lines as necessary are presented. Provided is the “Ensembl Gene ID” and associated “Gene Symbol” and “Description”, the chromosome location (“Chromosome” and “Strand” of the gene; the “Start” and “End” of the motif), the Transfac “Matrix ID” that matched the motif, the corresponding “Genomic sequence”, its position relative to the Transcription Start Site (“−10000/−5000”, “−5000/−2000”, “−2000/−1000”, “−1000/0”, “0/+200”, “+200/+400”, “+400/+1000” and “+1000/+2000”; labelled “0” or “1”), the “Cross-species conservation score” and the “Orientation” of the motif (+ or −). Column D indicates those genes that are “Predicted as “Somatic” with a proximal cARE”. If such a gene harbours several putative AREs, then only that (those) identified in the proximal region is (are) bolded.
(XLS)
