# Supplementary figures and images for: Profiling of Androgen Response in Rainbow Trout Pubertal Testis: Relevance to Male Gonad Development and Spermatogenesis
Source: PLoS One. 2013 Jan 3;8(1):e53302. doi: 10.1371/journal.pone.0053302 (PMC3536770; doi:10.1371/journal.pone.0053302)

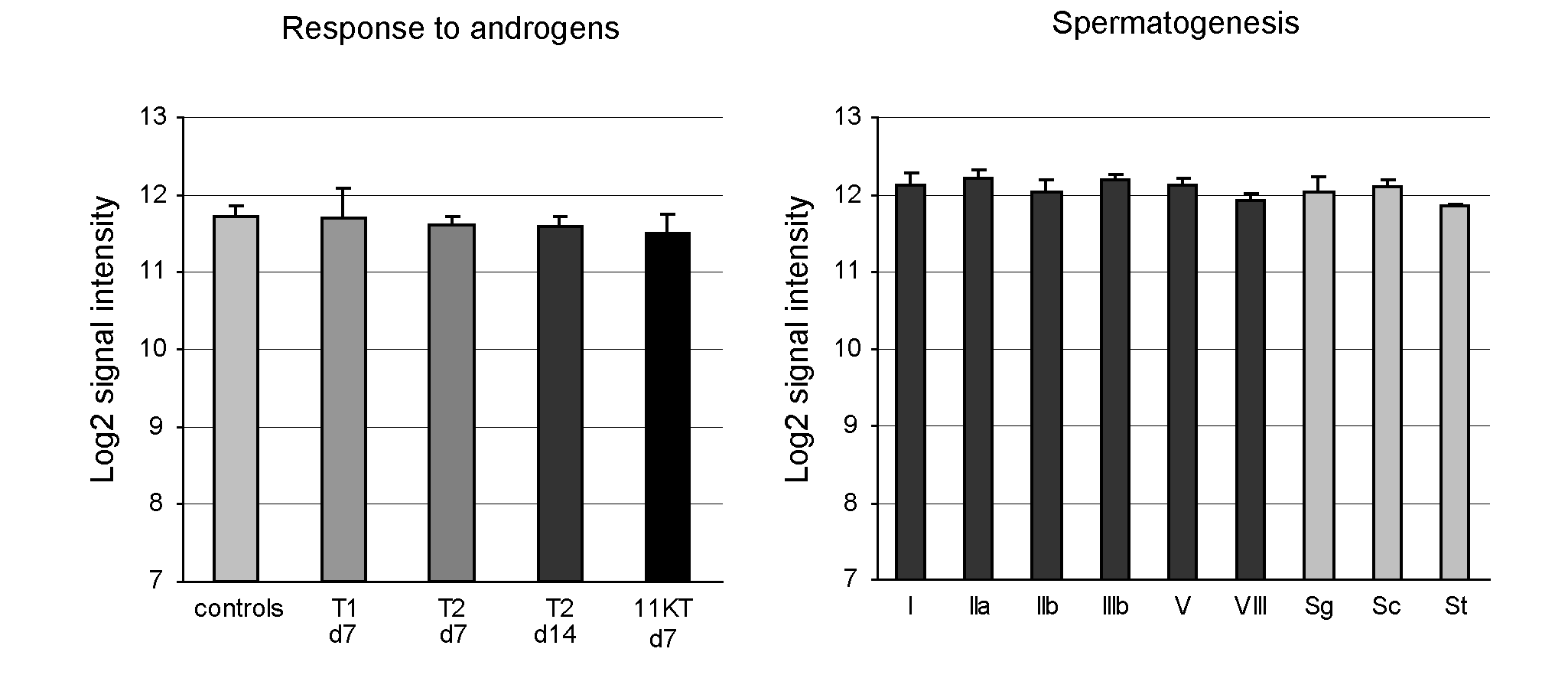

Supplement: Figure S1 — Expression profile of the reference gene rps15 in microarray datasets. Expression of rsp15 (clone 1RT58B15_B_A08) as measured in androgen supplementation and spermatogenesis datasets. Histograms represent means ± standard deviation of Log2-transformed signal intensities. Cont correspond to untreated control animals. T1 and T2 correspond to animals supplemented with testosterone implants of 0.1 and 0.2 mg, respectively. 11KT indicates animals treated with 11-ketotestosterone implants of 0.25 mg. Day 7 and Day 14 indicate 7 and 14 day post-implantation, respectively. Roman numbers (I–V and VIII) indicate testicular developmental stages. Sg = spermatogonia. Sc = spermatocytes. St = spermatids. (TIF) [file pone.0053302.s001.tif]

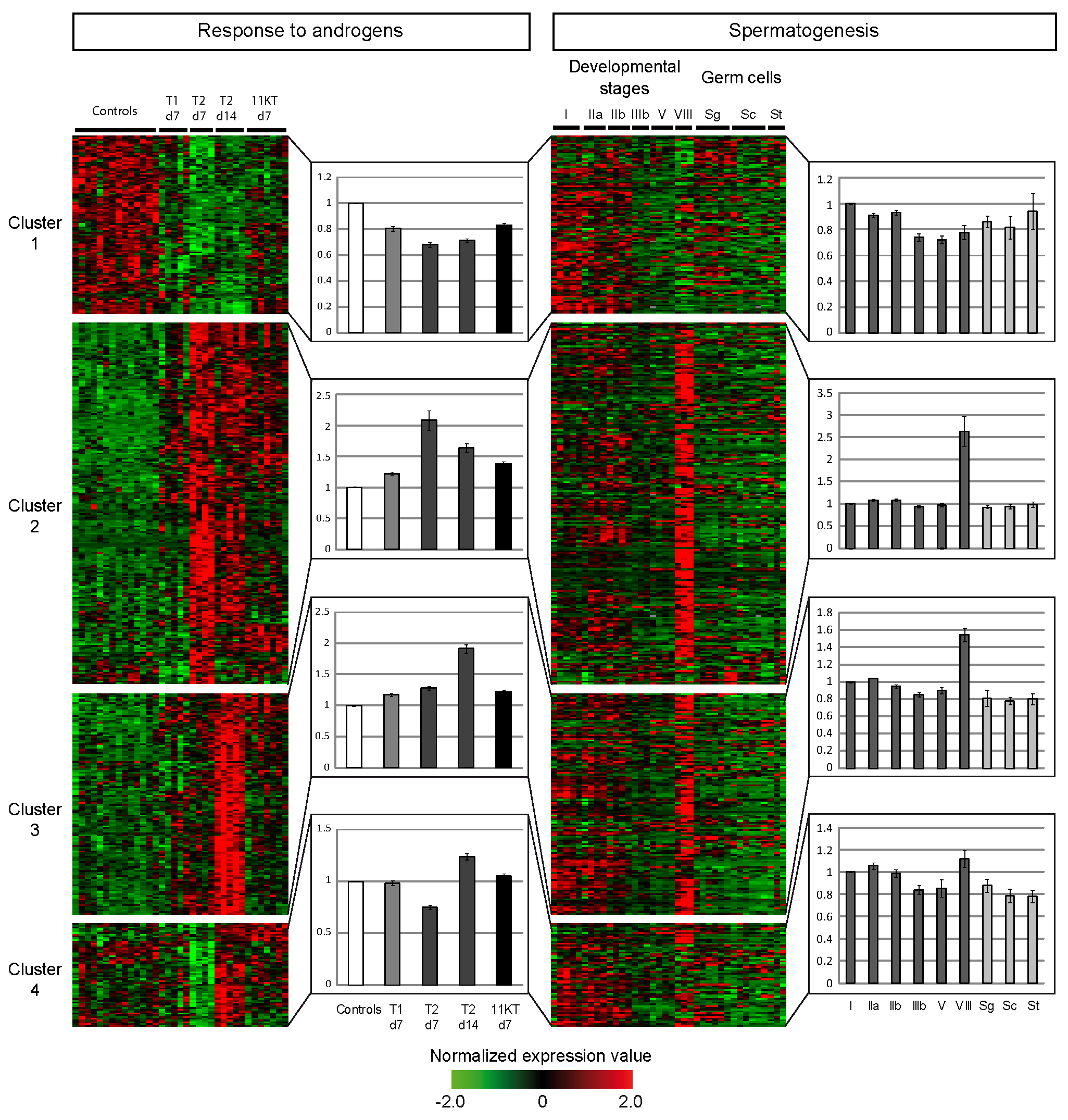

Supplement: Figure S2 — Changes in testicular gene expression following androgen supplementation. Heatmap representation of 418 androgen-responsive genes (485 clones) in the trout testis. After statistical filtration, co-expressed transcripts were classified into 4 clusters (1–4) using the PAM algorithm. For these genes, we present expression signals obtained: - in testes from controls and androgen-supplemented animals, and - in testes at various developmental stages and enriched fractions of isolated germ cells. Each line represents the expression signal of a single clone and each column is a sample. Normalized expression values are shown according to the scale bar while histograms represent averaged fold changes to the control (androgens data) or to satge I (spermatogenesis data) ± SEM for each cluster. T1 and T2 correspond to animals supplemented with 0.1 and 0.2 mg testosterone implants, respectively; 11KT indicates animals treated with 0.25 mg 11-ketotestosterone implants; d7 and d14 correspond to animals treated for 7 and 14 days, respectively. The gene annotation of each cluster is accessible in the searchable File S1. (TIF) [file pone.0053302.s002.tif]

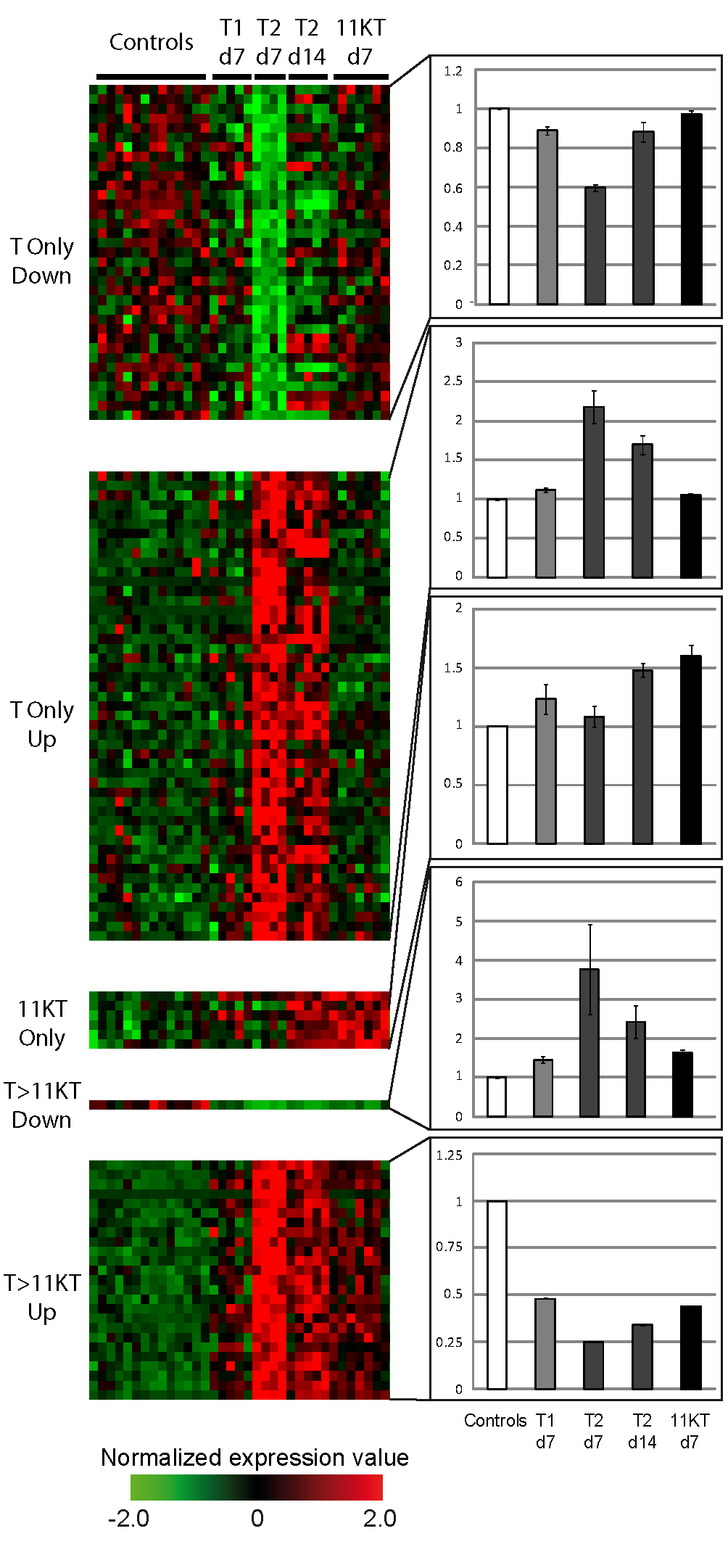

Supplement: Figure S3 — Expression of genes altered differently by testosterone and 11-ketotestosterone treatments. Heatmap representation of 103 androgen-responsive genes (116 clones) which regulation by testosterone and 11-ketotestosterone differs statistically. Genes are displayed according to their response to testosterone only (T only; Up- or Down-regulated), to 11-ketotestosterone only, or to their greater response to testosterone (T >11KT; Up or Down regulated). Each line represents the expression signal of a single clone and each column is a sample. Normalized expression values are shown according to the scale bar while histograms represent averaged fold changes to the control ± SEM for each cluster. T1 and T2 correspond to animals supplemented with testosterone implants of 0.1 and 0.2 mg, respectively; 11KT indicates animals treated with 11-ketotestosterone implants of 0.25 mg; d7 and d14 correspond to animals treated during 7 and 14 days, respectively. The gene annotation of each cluster is accessible in the searchable File S1. (TIF) [file pone.0053302.s003.tif]

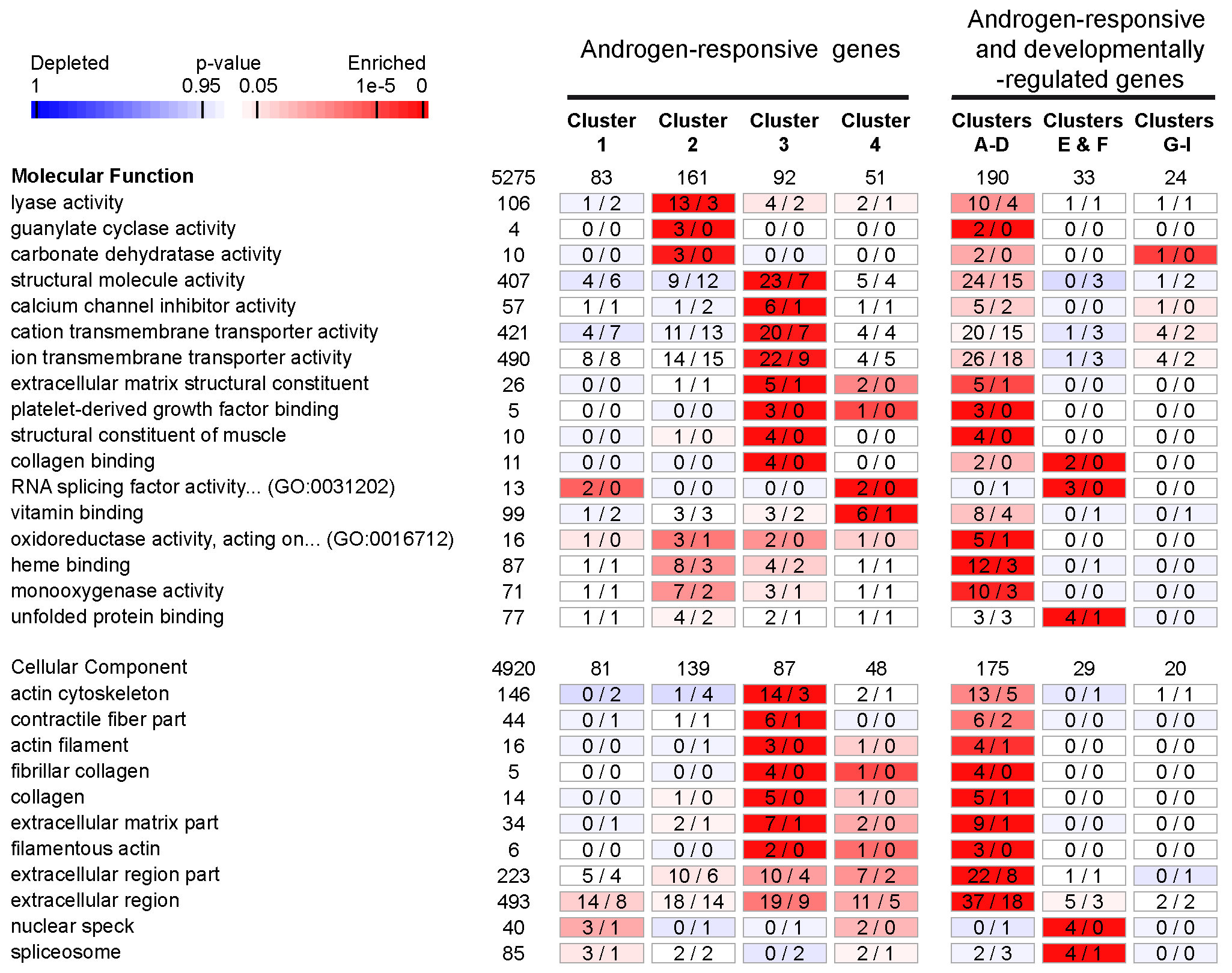

Supplement: Figure S4 — Enriched molecular functions and cellular components GeneOntology terms among testicular androgen-responsive genes. Over-represented “biological process” terms from the GeneOntology (GO) were identified in the 4 expression clusters of androgen-responsive genes (1 to 4) as well as in 3 groups of androgen-regulated genes that exhibit “somatic” (A to D), “spermatogonial” (E and F) and “germline” (G to I) expression profiles during spermatogenesis. Rectangles indicate the observed (left) and expected (right) numbers of genes bearing the corresponding GO term whereas the number of genes exhibiting this GO term on the entire microarray is given on the left. Only GO terms with a p-value of ≤10−6 and for which at least 3 non-redundant genes belonged to the cluster were considered as statistically-enriched. To avoid redundancy between closely related terms an Ontology Specific Information Rate (OSIR) cutoff of ≥0.95 was selected [27]. Bolded numbers indicate a statistical enrichment for a given GO term according to the scale bar. (TIF) [file pone.0053302.s004.tif]

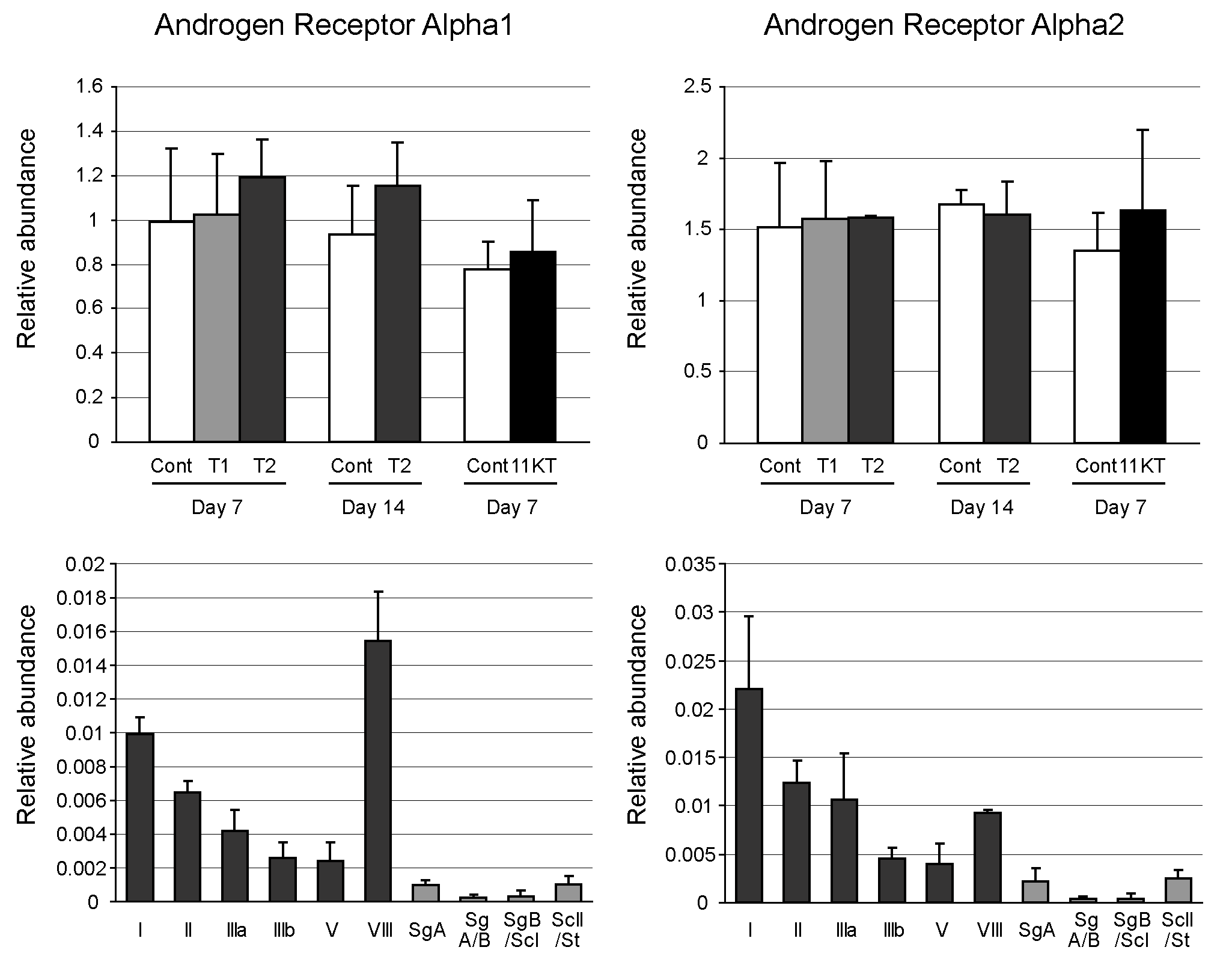

Supplement: Figure S5 — Expression profiles of arα1 and arα2. Expression of arα1 and arα2 was measured by qPCR and normalised to rps15 expression levels. Cont corresponds to untreated control animals. T1 and T2 correspond to animals supplemented with testosterone implants of 0.1 and 0.2 mg, respectively. 11KT indicates animals treated with 11-ketotestosterone implants of 0.25 mg. Day 7 and Day 14 indicate 7 and 14 day post-implantation, respectively. Roman numerals (I-VIII) indicate developmental stages. SgA/B = Type A/B spermatogonia; ScI/II = primary/secondary spermatocytes; St = spermatids (TIF) [file pone.0053302.s005.tif]

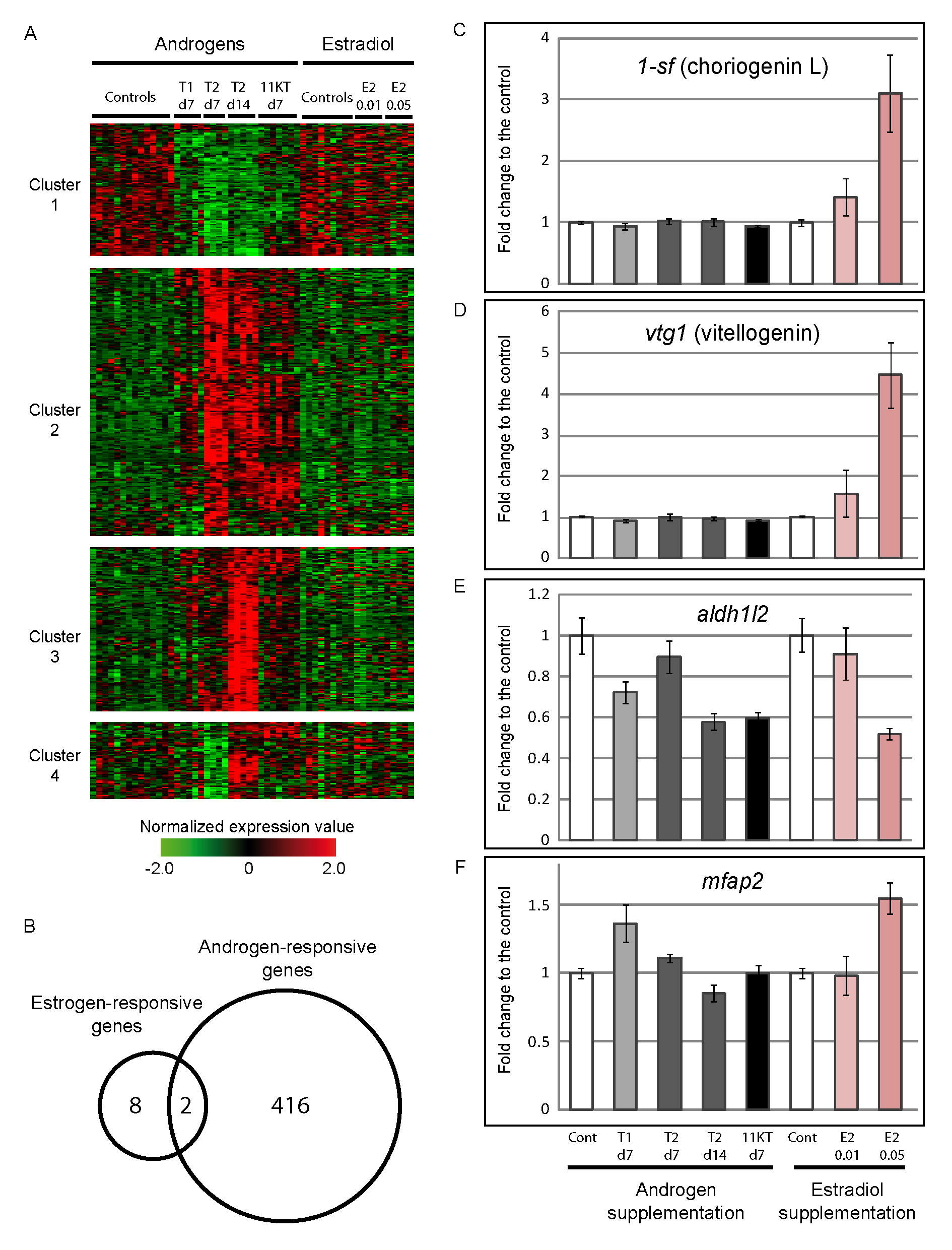

Supplement: Figure S6 — Androgen- versus estrogen-responsive genes. A: Heatmap representation of the expression of all the androgen-responsive genes according to the androgen-supplementation experiment (this study) as well as to the estradiol-supplementation experiment (unpublished data). Normalized expression data are displayed according to the scale bar. B: Venn diagram showing the overlap between androgen-responsive genes (this study) and estradiol-responsive genes (unpublished data). C-F: Expression profiles (as determined by microarray analysis) for two estradiol-responsive genes (choriogenin in C, vitellogenin in D) and for two estradiol- and androgene-responsive genes (aldh1l2 in E, mfap2 in E). Histograms represent fold changes to the control ± SEM. (TIF) [file pone.0053302.s006.tif]
