# Supplementary material for: Profiling of Androgen Response in Rainbow Trout Pubertal Testis: Relevance to Male Gonad Development and Spermatogenesis
Source: PLoS One. 2013 Jan 3;8(1):e53302. doi: 10.1371/journal.pone.0053302 (PMC3536770; doi:10.1371/journal.pone.0053302)
Supplement: Table S1 — Sequences of primers used in qPCR experiments. The clone name, the corresponding gene symbol and the sequence of forward and reverse primers (5′-3′) used for qPCR measurements are indicated. (DOC) [file pone.0053302.s007.doc]

Sequences for primers used in qPCR experiments

| **Gene Symbol** | **Forward primer (5’-3’)** | **Reverse primer (5’-3’)** |
| --- | --- | --- |
| Rps15 (reference) | CCTGGGGGAGTTCTCTATCACCT | GGGATGAAACGGGAAGAATGTGT |
| Amh | GGGAATAACCATGCTATCCTGCTTAA | CTCCACCACCTTGAGGTCCTCATAGT |
| Angptl7 | CTTTGATTCCTAACTCACTCCAACC | GGTCCAGTCTGATTCTTTAGCGG |
| Ara1 | GCTCGCTCACGGTCTTATCA | CATGTGCTGGGGGTTTGTAGA |
| Ara2 | CTCGTCTCATGTCATCCCAGC | AATATGGACCGAGGCAGGGT |
| Bty | CAGACAGGTATTGAAGAATGGA | AGACTACAAGAAGGGAGAGG |
| Cld11 | TTCCTTTCACAATAGTCCCACAG | CAGTCCACAGCCCAAATAGTC |
| Clu | AGCAGAATGTTCAGGATGAC | TTATTCAGTTCTTACAGCAGGACGA |
| Cxcl14 | GTAAGAGGAAAGGAACATAGGG | TCTTCATCACATTCGCTAGAC |
| Dmrt1 | GGACACCTCCTACTACAACTTCTA | GTTCGGCATCTGGTATTGTTGGT |
| Il13ra2 | GGGTGCTTCAAATAAGTGTG | AGAGAACCAGTAGTATAGCCT |
| Inhba | AGGGCAAGGTGAACATACAG | CCTCGTGTCCACCATCTTCTC |
| Inha | CCTGGTTGTCGAGGGAGGATTG | CCAGCTCTGACTCTACCTGTGAT |
| Morn3 | CTCCATTCTCCCTCATAGCA | TAGCAAACTCCTGTCTGTAACC |
| Noc2l | ATGATTGAATGTACGGAAAGATGGG | AGAGGGAGACGAGGACTTGG |
| Rsph3 | CCAACACTGCTCTTGCCACG | TACACCACGCTGAGGGAACAC |
| Sox9a | GTATTTCCAGTTCTTTCAGCCA | TTTGCTATCTAGTTGTGTACGG |
| Sox9b | AGCAGCAGTTGGATTCTAAAGTC | ACACTTCTCCTGTTCGTCTG |
| Star | GAGTTGTTAGGGCAGAGAAC | CAACCCTTTAAATCTATGCTTA |
| Tbx1 | CTTCGGCTACTAGTGCTGTGGAA | CAACCTCCCAACCTTCTAACCTC |
| Zpacp | CCCTATCACAACACCTCCGA | CCCAACTCAAGCACAAACACTG |
